# A Lead (II) 3D Coordination Polymer Based on a Marine Cyclic Peptide Motif

**DOI:** 10.3390/molecules18054972

**Published:** 2013-04-26

**Authors:** Subrata Chakraborty, Pooja Tyagi, Dar-Fu Tai, Gene-Hsiang Lee, Shie-Ming Peng

**Affiliations:** 1 Department of Chemistry, National Dong-Hwa University, Hualien 974, Taiwan; 2 Department of Chemistry, National Taiwan University, Taipei 106, Taiwan

**Keywords:** natural product, cyclic peptide, lead, coordination polymer, crystal structures, macrocyclic chemistry

## Abstract

The crystal structure of a naturally occurring cyclic tetrapeptide *cyclo*(Gly-l-Ser-l-Pro-l-Glu) [*cyclo*(GSPE)] was obtained. The conformation of synthesized *cyclo*(GSPE) fixes the coordination to lead ion in a 1:1 ratio. This *cyclo*(GSPE)-Pb complex was constructed as an asymmetric 3D network in the crystalline state. The polymerization of a heavy metal ion with a rigid asymmetric cyclic tetrapeptide represents the first example of a new class of macrocyclic complexes.

## 1. Introduction

In the area of biomedicinal chemistry much interest has been generated in the design of metal-organic frameworks (MOFs) [1,2]. Due to their chemical variety, biocompatibility and ability to assemble spontaneously, peptides can form excellent simple nanotubes [3,4,5]. Some metal-peptide frameworks (MPFs) [6] have been reported, but very few of them have been characterized by crystallography [6,7]. The size and conformation of cyclic peptides [8,9] and their metal binding side chains provide a novel tool for construction of discrete metal-assembled supermolecules with unique chemical and physical properties. Cyclic tetrapeptides (CTPs) [10,11,12,13,14,15] also possess a definite inner nanocavity of a constrained nature and hence, generated highly entropic advantages in molecular recognition with the potential to confer chirality and biological activity on these compounds [16,17,18]. Compared with the d- and f-block metals, Pb^2+^ ion has a larger radius, variable coordination numbers and unique prospects for the construction of novel polymeric or multi-dimensional supramolecular networks [19,20,21,22,23,24,25]. The chemistry of lead has drawn a lot of interest for its wide applications in fields such as fuel additives, batteries, oil refining and paint manufacturing, but also in biological systems, where Pb metal has been found to interact with many amino acids, peptides and proteins: these binding preferences of Pb(II) have provided the inspiration for the design of a selective chelating therapy agent [26,27,28,29,30,31].

*Cyclo*(Gly-l-Ser-l-Pro-l-Glu) [*cyclo*(GSPE), **1**] has metal binding sites on the side chains (serine hydroxyl and glutamic carboxylate group) and the carbonyl groups. It was first isolated from the *Ruegeria* strain of marine bacteria [32] and was found to possess moderate antibacterial activity against *Bacillus subtilis*. Its structure was elucidated on the basis of 1D and 2D NMR data, followed by the determination of absolute configuration [33]. However, a crystal structure has not been reported. Due to strain of the twelve member ring, binding to metal ions by carbonyls is more difficult than by side chains. Keeping in mind the combination of hydroxyl and carboxylato groups and from a study of natural CTPs-metal complexes, *cyclo*(GSPE) **1** was selected as a linking ligand to investigate the coordination chemistry of Pb(II) ion. Here the crystal structure of *cyclo*(GSPE) and {[*cyclo*(Gly-l-Ser-l-Pro-l-Glu)Pb(NO_3_)]·2H_2_O}_n_ **2** are reported for the first time. Further {[*cyclo*(Gly-l-Ser-l-Pro-l-Glu)Pb(NO_3_)]·2H_2_O}_n_ **2** was constructed as an asymmetric 3D network in the crystalline state. Coordination relied on the prearranged conformation of synthesized *cyclo*(GSPE) **1**. The polymerization of a heavy metal ion with a rigid asymmetric cyclic tetrapeptide represents the first example of a new class of complexes. 

## 2. Results and Discussion

### 2.1. Synthesis of *cyclo*(GSPE) **1** and {[cyclo(Gly-l-Ser-l-Pro-l-Glu)Pb(NO_3_)]·2H_2_O}_n_ **2**

*Cyclo*(GSPE) **1** was synthesized by using standard solution-phase peptide coupling protocols. Its spectral data were identical with those of natural **1** [32,33]. Crystals were prepared for the first time by slow cooling to room temperature in water. The synthesized peptide **1** was subjected to complexation with Pb(II). By heating an aqueous solution of the peptide **1** and the lead(II) salt in a 1:1 ratio, the {[*cyclo*(Gly-l-Ser-l-Pro-l-Glu)Pb(NO_3_)]·2H_2_O}_n_ **2** complex was obtained. Crystals were prepared by slow cooling to room temperature and resulted in a 3D coordination polymer. Several coordination polymers of lead with a variety of synthetic organic ligands have been reported earlier [34,35,36,37,38]. The structure of macrocyclic complex **2** is the first solved marine *cyclo*(GSPE) **1** lead complex. 

### 2.2. Characterization of {[*cyclo*(Gly-l-Ser-l-Pro-l-Glu)Pb(NO_3_)]·2H_2_O}_n_ **2**

*NMR Spectra*. Interaction between *cyclo*(GSPE) **1** with Pb^2+^ was first examined by ^1^H-NMR after reaction with Pb(NO_3_)_2_ (1.0 equiv) in water at pH 5.0 and 70 °C for 4 days. The NMR spectrum of the complex **2** showed the appearance of new multiplet (δ = 4.19–4.26) and triplet (δ = 4.76) peaks for glutamic acid and the serine α-protons in place of the doublet (δ = 4.08–4.10) and multiplet (δ = 4.81–4.85) peaks of the free peptide, respectively (Figure S3). These NMR signal changes only in the side chain protons clearly indicated their binding with Pb^2+^ ions outside of the cyclic peptide ring. 

*Mass Spectra*. MALDI-TOF mass spectrometry on the reaction product with 1.0 equivalents of Pb^2+^ ions showed interaction with the peptide, with peaks at *m/z* 577.69, 595.71 and 719.69 corresponding to [M+Pb–H]^+^, [M+Pb+H_2_O–H]^+^ and [M+Pb+2NO_3_+H_2_O–H]^+^, respectively. This was further confirmed by HR-ESI mass spectrometry (Figure S4, Figure S5, Figure S6 and Figure S7). All the assignments showed good agreement between the observed and calculated isotopic distributions.

*IR Spectra.* The IR spectrum of macrocycle **1** showed a characteristic ν(C=O) absorption band for the –COOH group at 1,718 cm^−1^, which vanishes in the case of metal complex **2**. For the free peptide the ν_as_(COO) and ν_s_(COO) stretching vibrations of the carboxylate group appear at 1,545 and 1,444 cm^−1^ respectively, while for the lead complex **2**, the first absorption region became broad and the second was obscured by the nitrate ion absorption, which displays a quite intense band at 1,384 cm^−1^ (Figure S8 and Figure S9).

### 2.3. Crystal Structures

#### 2.3.1. Crystal Structure of CTP **1**

X-ray crystallographic studies showed a crystal structure of macrocycle **1** with a *cis-trans-cis-trans* (two *transoid* amide bonds between Gly-Ser, Pro-Glu and two *cisoid* amide bonds between Ser-Pro, and Glu-Gly) peptide bond, a sequence closely associated with biological activity. A summary of the crystal data and refinement results is listed in Table 1.

**Table 1:** Selected crystallographic data of **1** and **2**.

	1	2
Empirical formula	C_15_H_22_N_4_O_7_	C_15_H_25_N_5_O_12_Pb
Formula weight	370.37	674.59
Temperature	296(2) K	296(2) K
Wavelength	0.71073	0.71073
Crystal system, space group	Monoclinic, P2(1)	Monoclinic, P2(1)
Unit cell dimensions		
a (Å)	4.9855(7)	5.0532(3)
b (Å)	17.257(3)	17.7774(11)
c (Å)	9.7830(14)	11.8317(5)
α (°)	90.00	90.00
β (°)	104.064(3)	94.550(3)
γ (°)	90.00	90.00
Volume (Å^3^)	816.4(2)	1059.52(10)
Z, Calculated density (Mg/m^3^)	2, 1.507	2, 2.115
Absorption coef. (mm^−1^)	0.121	8.037
F(000)	392	656
Crystal size (mm)	0.13 × 0.08 × 0.05	0.44 × 0.09 × 0.05
θ range for data collection (°)	2.15-28.10	1.73-27.5
Limiting indices	−5≤ h ≤6, −22 ≤ k ≤ 22, −12 ≤ l ≤ 12	−6≤ h ≤ 6, −23 ≤ k ≤11, −14 ≤ l ≤ 15
Reflection collected/unique	7693/3848 [R(int) = 0.0395]	6993/3657 [R(int) = 0.0363]
Absorption correction	Multi-scan	Multi-scan
Max. and min. transmission	0.7456 and 0.6392	0.6990 and 0.1278
Refinement method	Full-matrix least-squares on F^2^	Full-matrix least-squares on F^2^
Data/restraints/parameter	3848/1/237	3657/1/298
Goodness-of-fit on F^2^	1.063	0.949
Final R indices [I > 2sigma(I)]	R_1_ = 0.0471, wR_2_ = 0.1071	R_1_ = 0.0278, wR_2_ = 0.0618
R indices (all data)	R_1_ = 0.0739, wR_2_ = 0.1292	R_1_ = 0.0317, wR_2_ = 0.0636
Larg. diff. peak and hole (e.Å^−3^)	0.214 and −0.222 e.Å^−3^	1.336 and −1.127 e.Å^−3^

The backbone of the cyclic peptide adopts a two-fold symmetric conformation which is very common for cyclic tetrapeptides containing alternate ‘*cis*’ and ‘*trans*’ peptide units and the two side chains (glutamic and serine) are away from the ring. The glutamic residue assumes the *gauche^−^* conformation to the main-chain amino group (*χ^1^* = −54.6°). In the prolyl residue, C^α^, C^β^, C^δ^ and N form an approximate plane with C^γ^ deviating from the plane by 0.56 Å towards the side of the carbonyl group. The torsion angles that define the conformation of the molecule are listed in Table 2. 

**Table 2:** Backbone and side-chain torsion angles (°) of **1**.

Angle	Gly	l-Ser	l-Pro	l-Glu
*Φ*	−98.2	−134.5	−84.3	55.1
*ψ*	−13.6	49.7	153.9	47.0
*ω*	−176.07	2.91	177.26	13.45
*χ^1^*			32.15	−54.65
*χ^2^*			−39.57	151.99
*χ^3^*			32.21	
*χ^4^*			−11.30	
*χ^5^*			−13.08	

**Figure 1:**
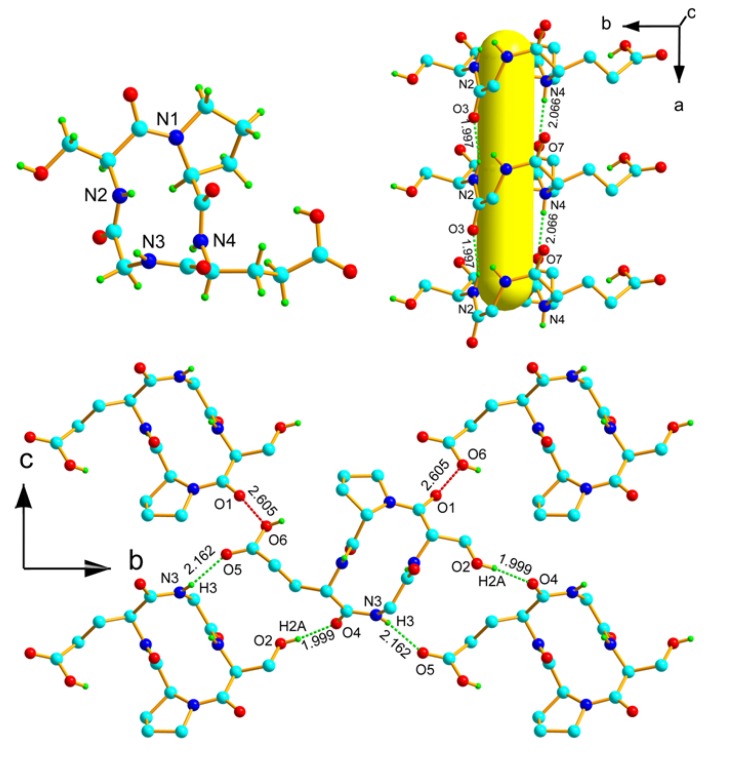
Thermal ellipsoid plot of *cyclo*(Gly-l-Ser-l-Pro-l-Glu) **1**. Color code: light gray, C; blue, N; red, O; dark gray, H.

The conformation of the cyclic tetrapeptide **1** is illustrated in the Figure 1 (top, left). The overall shape of the backbone is a distorted boat with the *cis* amide bonds at the two ends which could facilitate ion binding. 

Each discrete molecule of *cyclo*(GSPE) **1** is strongly hydrogen bonded to a neighboring molecule through the amide N-H of glutamic acid moiety to a carboxyl O-atom of proline (*d*_N-H···O_ 2.066 Å and *θ*_N-H···O_ 176.6°) moiety and amide N-H of serine to a carboxyl O-atom of a glycine moiety (*d*_N-H···O_ 1.997 Å and *θ*_N-H···O_ 145.3°), to form a 1D-chain along the a-axis. An oriented CTP nanotube (yellow) is thus assembled. The structure of this nanotube is expanded into a three-dimensional network (5.4 × 2.8 Å^2^), which is devoid of water molecules (Figure 1, top and right). 

The carboxylic acid group of glutamic acid and the hydroxyl group of serine are flanked in opposite directions and are hydrogen bonded to a neighboring 1D-chain to form an overall 3D-network. Each *cyclo*(GSPE) **1** molecule is surrounded by four neighboring molecules and two molecules are hydrogen bonded (between amide N-H of glycine moiety of neighboring molecule to carboxyl O-atom of glutamic acid moiety of the core molecule (*d*_N-H···O_ 2.162 Å and *θ*_N-H···O_ 154.1°), and hydroxyl H-atom of serine from neighboring molecule to O-atom of the amide group of the glutamic acid moiety (*d*_O-H···O_ 1.999 Å and *θ*_O-H···O_ 169.0°) of the core molecule. The remaining two neighboring molecules are linked to the core molecule through O···O interactions between the amide O-atom of the serine moiety to the O-atom of carboxylic acid group of glutamic acid (*d*_C-O···O_ 2.605 Å and *θ*_C-O···O_ 169.1°) moiety (Figure 1, bottom).

#### 2.3.2. Crystal Structure of Complex **2**

**Figure 2:**
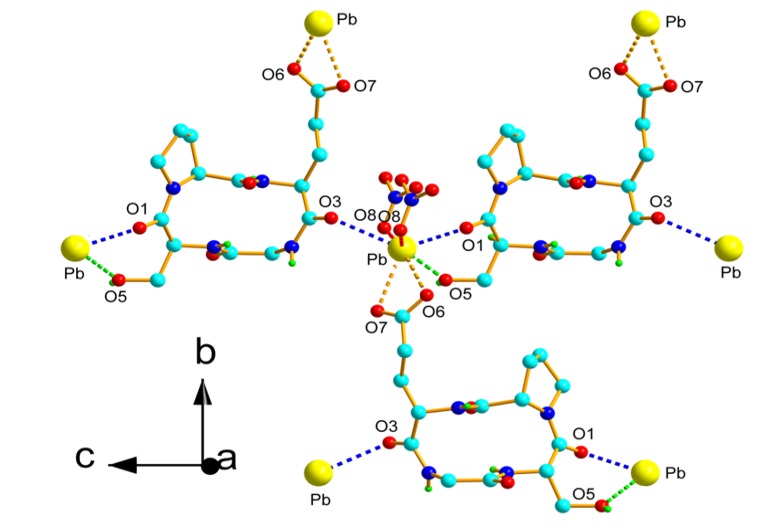
Crystal structure of **2**. Perspective view showing binding mode of *cyclo*(Gly-l-Ser-l-Pro-l-Glu) **1** to Pb(II) ion. Only hydroxyl O-H and amide N-H group hydrogen atoms are shown.

A single-crystal X-ray diffraction study reveals that complex **2** is a 3D-coordinated polymer, crystallized in the monoclinic system with *P* 2(1) space group. Crystallographic data of **2** are listed in Table 1. As shown in Figure 2, the asymmetric unit of **2** is composed of one Pb(II) center, one deprotonated *cyclo*(GSPE) ligand, one nitrate molecule and two lattice water molecules.

The Pb(II) center is in a seven coordination environment (Figure 2) with five oxygen atoms [O1(proline carbonyl), O3(glutamic carbonyl), O5(serine hydroxyl) O6 and O7(glutamic carboxylate)] of three distinct *cyclo*(GSPE) **1** ligands and two *μ*_1,1_- oxygen atoms (O8) of nitrate molecules from two EO bridges (joining it to a neighboring Pb atom). The torsion angles that define the conformation of the complex **2** are listed in Table 3.

**Table 3:** Selected bond lengths (Å) and angles (°) of **2**.

Pb-O1	2.489(4)	Pb-O3	2.833(5)
Pb-O5	2.475(5)	Pb-O6	2.463(6)
Pb-O7	2.644(6)	Pb-O8	2.571(5)
Pb-O8	2.755(5)		
O6-Pb-O5	73.42(19)	O6-Pb-O1	80.12(17)
O5-Pb-O1	69.53(16)	O6-Pb-O8	71.78(19)
O5-Pb-O8	135.80(18)	O1-Pb-O8	78.36(16)
O6-Pb-O7	50.55(17)	O5-Pb-O7	78.39(19)
O1-Pb-O7	127.25(17)	O8-Pb-O7	99.17(19)
O6-Pb-O8	145.03(18)	O5-Pb-O8	75.99(18)
O1-Pb-O8	104.61(16)	O8-Pb-O8	143.1(3)
O7-Pb-O8	106.82(18)	O6-Pb-O3	129.40(16)
O5-Pb-O3	143.21(16)	O1-Pb-O3	134.98(17)
O8-Pb-O3	80.86(17)	O7-Pb-O3	95.17(17)
O8-Pb-O3	71.33(15)		

**Figure 3:**
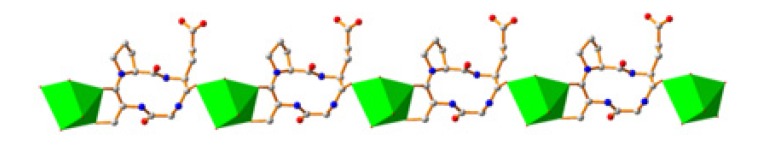
View of the 1D linear chain of **2** along [001] direction. Color code: green, Pb; gray, C; blue, N; red, O.

The geometry around the Pb center appears holodirected [39]. The bidentate carboxylate oxygen atoms (O6 and O7) provided the asymmetric chelation (Pb-O 2.463(6) and 2.643 (6) Å). The Pb-O bond distances (Table 3) are in the range of 2.463(6)–2.833(5) Å and the O-Pb-O bond angles vary from 50.55(17)–145.03(18)° and are comparable to those reported earlier for lead-oxygen donor complexes [24,40,41,42]. 1D linear chain around the Pb center is formed *via* carbonyl and hydroxyl oxygen atoms of two distinct *cyclo*(GSPE) ligands along [001] direction (Figure 3). 

**Figure 4:**
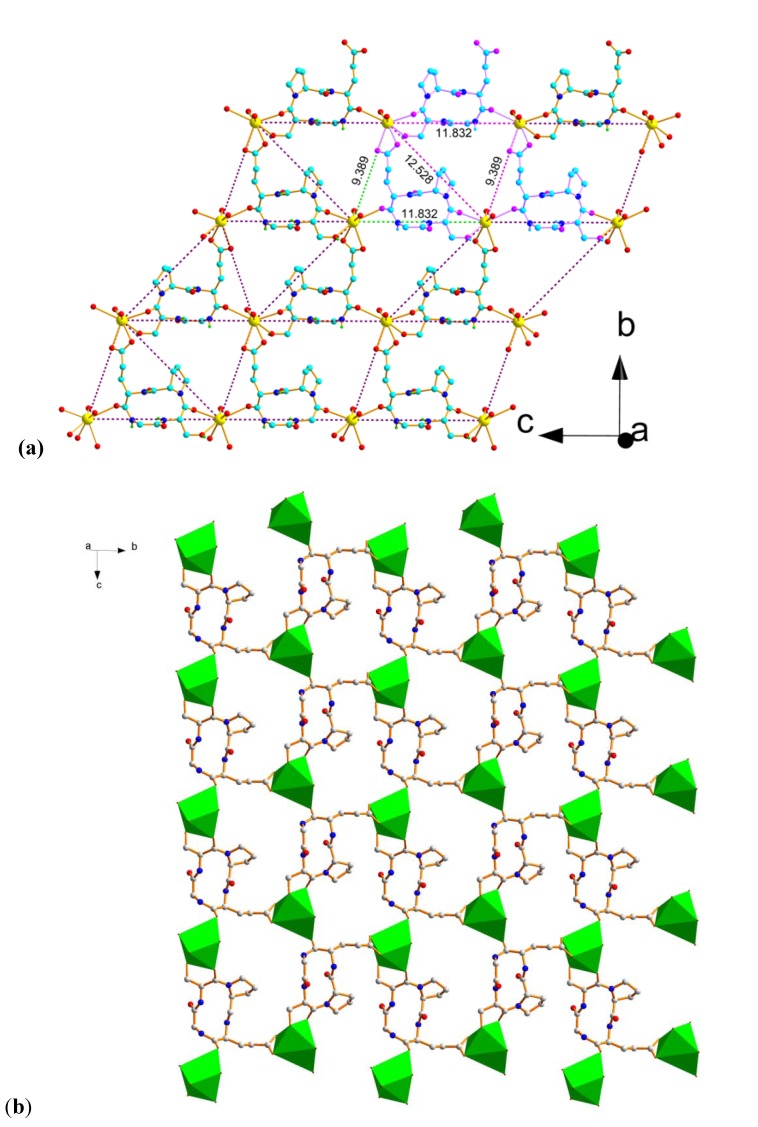
(**a**) Perspective view of 2D-network along a-axis. Color code: yellow, Pb; cyan, C; blue, N; red, O. (**b**) 2D sheet of **2** in the bc plane. Color code: green, Pb; gray, C; blue, N; red, O.

**Figure 5:**
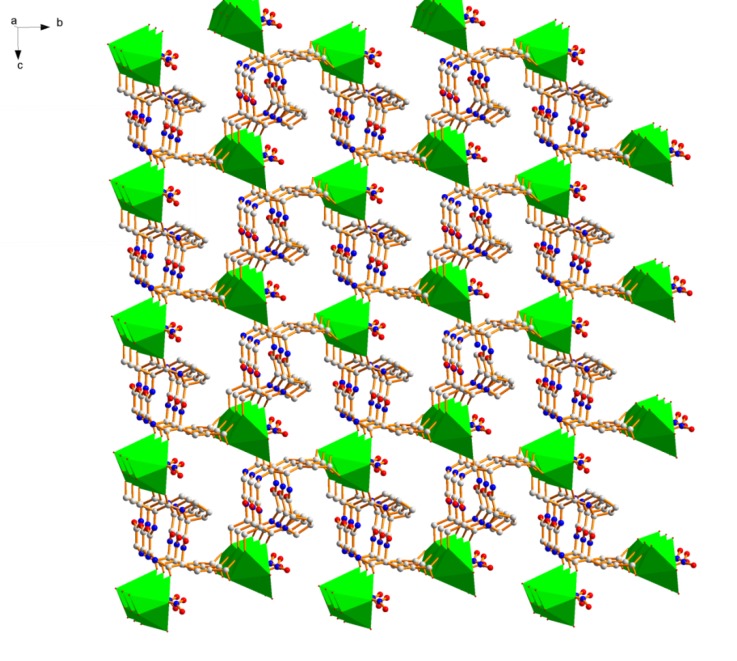
Polyhedral view in three-dimensional structure of **2**. Color code: green, Pb; gray, C; blue, N; red, O.

Three molecules of *cyclo*(GSPE) **1** binds with three Pb(II) ions to form one cyclic ring and the same unit repeats along b-axis to form a 2D layer structure (Figure 4a,b). When viewed along the [100] direction, these sheets are further cross-linked by NO_3_^−^ molecules giving rise to a three-dimensional structure. The size and the shape of the CTP nanotube remains unchanged (Figure 5).

**Figure 6:**
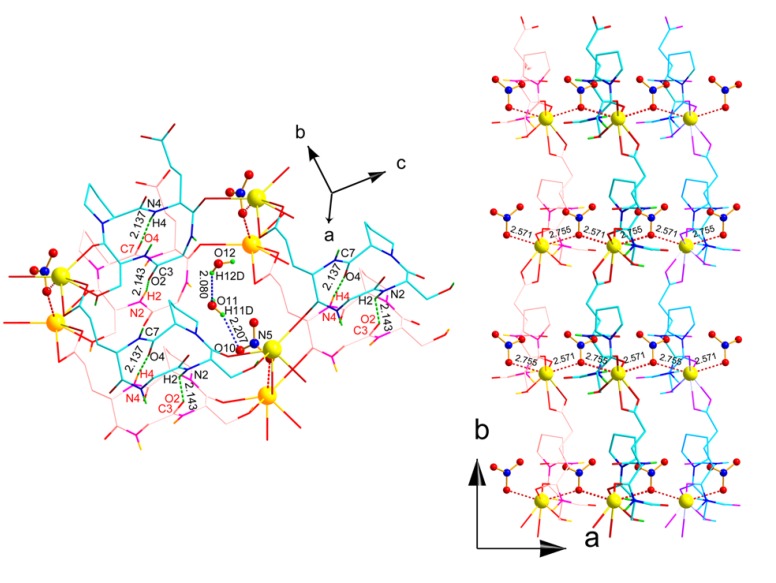
Perspective view of hydrogen bonded (between amide N-H and carbonyl O-atom) cage (*left*) of two layer and each cyclic layer formed by three *cyclo*(Gly-l-Ser-l-Pro-l-Glu) **1** molecule coordinated to three Pb(II) ions. Between two layers, nitrate anions are weakly coordinated to two Pb(II) ions of each layer (*right*). Out of three nitrate anion, one nitrate anion lying inside the cage is further coordinated to a water molecule and the remaining two nitrate anions lying inside two neighboring cages. For clarity, each layer is differentiated from neighboring layer by different color.

Two adjacent Pb(II) ions in **2** are bridged by one oxygen atom from *μ*_1,1_-bridging NO_3_^−^ molecule (Figure 6) to build a dinuclear unit with a Pb···Pb distance of 5.053(0) Å. Of three coordinated nitrates to Pb(II), one nitrate faces the cavity of the cage (Figure 6, left) and the remaining two nitrate anion lie in neighboring cavities. The coordinated nitrate facing the cavity of the cage interact with one (out of two) water molecule inside cage (*d*_O11-H11···O10_ 2.207 Å, *θ*_O11-H11···O10_ 155.7(53)°). 

### 2.4. Discussion

There is no report on the solid state structure of a natural (12-membered) cyclic tetrapeptide with a metal ion. The molecular structure of the *cyclo*(GSPE)-Pb complex 2 is unique as all the complexes reported to date are with higher cyclic peptides or β-cyclic tetrapeptides and are sandwich complexes [43,44,45]. Structure **2** showed that conformation of **1** remains *cis-trans-cis-trans* and only the side chains and ring carbonyls are pointing towards the Pb metal for binding and, hence, represent an example of a metal complex bound with both a cyclic skeleton and functional groups in side chains. Unlike linear peptides, the retention of conformation of **1** is attributed to the highly constrained nature of CTPs. The rigidity of cyclopeptide backbones ensures the formation of nanotubes [46]. The inability to adjust their conformation makes CTP-metal complexes rare. However, once it formed, the resulting CTP-metal complexes **2** will preserve the nanotubes. 

## 3. Experimental

### 3.1. Materials and Physical Measurements

All reagents were commercially available (Aldrich, Saint Louis, MO, USA or Merck, Darmstadt, Germany) and used as supplied. Solvents were dried by standard procedures. The NMR spectra were recorded on a Bruker DRX 400 (^1^H at 400.13 MHz, ^13^C at 100.03 MHz) spectrometer (Bruker Daltonik, Bremen, Germany). MALDI TOF was performed on a Bruker Autoflex MALDI-TOF mass spectrometer (Bruker Daltonik, Bremen, Germany). High-resolution electrospray ionization mass spectrometry (ESI-MS) was performed on a Shimadzu-LCMS-IT-TOF mass spectrometer (Shimadzu, Kyoto, Japan). Elemental analysis for C, H, N was performed on VarioEL-III elementar analyzer (Elementar, Hanau, Germany). Infrared spectra were recorded on a PerkinElmer Spectrum one FT-IR spectrometer (PerkinElmer, Shelton, CT, USA) using KBr pellets (4,000–400 cm^−1^). 

### 3.2. Sythesis

*Cyclo(Gly-l-Ser-l-Pro-l-Glu)* (**1**). Linear peptide Boc-GS(OBn)PE(OBn)_2_ was synthesized by using standard solution-phase peptide coupling protocols starting from Fmoc-L-proline. Subsequently, regioselective enzymatic hydrolysis of the α-benzyl ester on glutamate was achieved [47]. The linear precursor was then activated with pentafluorophenol and cyclized with pyridine to form dibenzyl protected CTP [48]. Deprotection of the benzyl group finished the synthesis of **1**. The overall yield was 19%. IR (KBr, cm^−1^): 3459 ν(NH), 3236 ν(OH vs –CH_2_OH), 3066 ν(CH), 2990 2961 ν(CH_2_), 1718 ν(C=O vs -COOH), 1663 1646 1615 ν(C=O vs amide), 1545 ν_as_(COO), 1444 ν_s_(COO). ^1^H-NMR: (D_2_O)* δ* 1.60–1.89 (m, 3H), 1.93–2.17 (m, 3H), 2.20–2.38 (m, 2H), 3.36–3.54 (m, 2H), 3.57–3.65 (m, 1H), 3.77–3.82 (m, 1H), 3.86–3.97 (m, 2H), 4.08–4.10 (d, 1H, *J* = 8 Hz), 4.35–4.37 (d, 1H, *J* = 8 Hz), 4.80–4.82 (m, 1H). MALDI-TOF: *m/z* 371.35 [M+H]^+^, ESI-MS: [M−H]^−^ calculated *m/z* 369.1488, obtained *m/z* 369.1426.

*{[cyclo(Gly-l-Ser-l-Pro-l-Glu)Pb(NO_3_)]·2H_2_O}_n_* (**2**). Pb(NO_3_)_2_ (4.5 mg, 13.5 μmol) was dissolved in water (1 mL). *cyclo*(GSPE) **1** (5.0 mg, 13.5 μmol) was dissolved in water (1 mL) and added to the metal solution. The pH of solution was adjuted to 5 with diluted HNO_3_ solution. The reaction mixture was stirred at RT for 10 minutes and then at 70 °C for 4 days. Crystals suitable for X-ray diffraction were obtained from saturated solution by slow cooling. Anal. Calc. for C_15_H_25_N_5_O_12_Pb (674.6): C 26.70, H 3.73, N 10.38, O 28.46. Found: C 26.23, H 2.94, N 10.64, O 28.17. IR (KBr, cm^−1^): 3433, ν(NH); 1645, ν(C=O vs amide); 1384, ν(NO_3_). ^1^H-NMR: (D_2_O) 1.86–1.99 (m, 3H); 2.04–2.31 (m, 3H); 2.35–2.53 (m, 2H); 3.45–3.55 (m, 1H); 3.59 (s, 1H); 3.65–3.71 (m, 1H); 3.73–3.81 (m, 1H); 3.82 (s, 1H); 3.87–4.00 (m, 1H); 4.19–4.26 (m, 1H); 4.38–4.45 (m, 1H); 4.76 (t, 1H, *J* = 5.88 Hz). MALDI-TOF: *m/z* 577.69 [M+Pb–H]^+^ , 595.71 [M+Pb+H_2_O–H]^+^ and 719.69 [M+Pb+2NO_3_+H_2_O–H]^+^. ESI-MS: [M+Pb–H]^+^ calculated *m/z* 577.1177, obtained *m/z* 577.1183; [M+Pb+NO_3_–H]^+^ calculated *m/z* 639.1055, obtained *m/z* 639.1049; [M+Pb+2NO_3_–H]^+^ calculated *m/z* 701.0933, obtained *m/z* 701.0973; [M+Pb+2NO_3_+2H_2_O+H]^+^ calculated *m/z* 739.1301, obtained *m/z* 739.2925. 

### 3.3. X-ray Crystallography

Colorless prism-like crystals of **1** and rod-like crystals of **2** were obtained by slow solvent evaporation at room temperature, respectively. Crystals suitable for X-ray diffraction analysis were selected with size of 0.13 × 0.08 × 0.05 mm for **1** and 0.44 × 0.09 × 0.05 mm for **2**. Crystals were mounted on a glass fiber and used for data collection. For both compounds **1** and **2** diffraction data were preliminarily collected with a Bruker APEX-II CCD diffractometer using graphite monochromated Mo*K*α radiation (λ = 0.71073 Å). Absorption corrections for the area detector were performed with the program SADABS. Structures were solved by direct methods and were refined against the least-squares methods on *F2* with the SHELXL-97 package, incorporated in SHELXTL/PC V5. Anisotropic thermal factors were used only for all non-hydrogen atoms. 

CCDC 831318 and 831319 contains the supplementary crystallographic data for this paper. These data can be obtained free of charge via www.ccdc.cam.ac.uk/conts/retrieving.html (or from the CCDC, 12 Union Road, Cambridge CB2 1EZ, UK; fax: +44 1223 336033; e-mail: deposit@ccdc.cam.ac.uk).

## 4. Conclusions

We have developed the synthesis of a CTP *cyclo*(GSPE) **1**. The synthetic building blocks were coupled through hydroxyl, carboxylate and two amide bonds to lead ion and thus created water-soluble, coordination polymers (tris-CTPs) with well-defined three-dimensional structures. This is the first report of a naturally occurring CTP as the host for metal complexation. The CTP retains its conformation through the binding process with metals. The unique crystal structure of this rigid molecule suggests the formation of a designated complex with nanotube properties. 

## Supplementary Materials

Supplementary materials can be accessed at: http://www.mdpi.com/1420-3049/18/5/4972/s1.
