# Supplementary material for: A Lead (II) 3D Coordination Polymer Based on a Marine Cyclic Peptide Motif
Source: Molecules. 2013 Apr 26;18(5):4972–85. doi: 10.3390/molecules18054972 (PMC6270303; doi:10.3390/molecules18054972)

Supporting Information

**Figure S1**. ^1^H-NMR:(D_2_O, 400 MHz) *Cyclo*(glycyl-L-seryl-L-prolyl-L-glutamyl) (compound **1**).


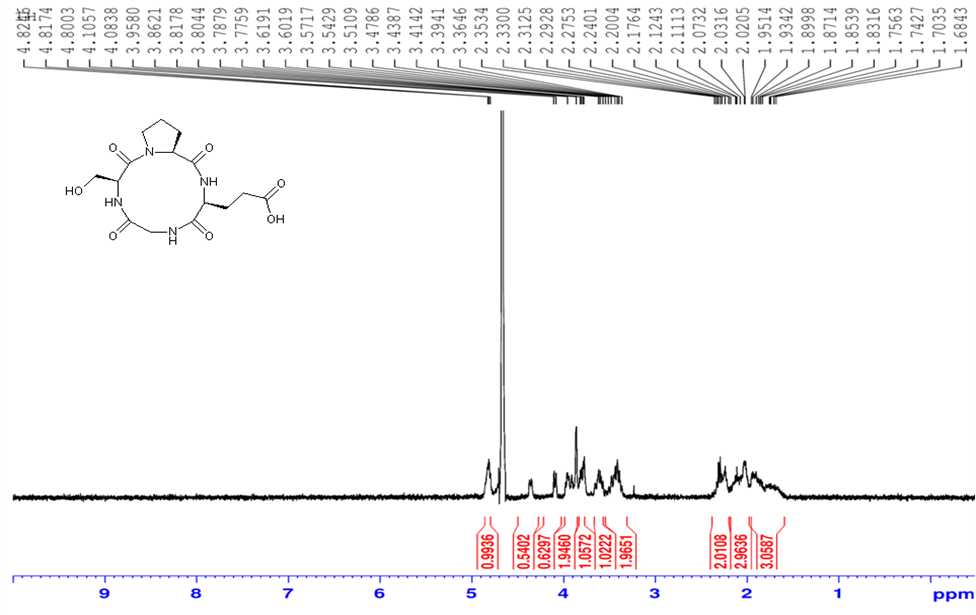


**Figure S2**. ^1^H-NMR:(D_2_O, 400 MHz) [*cyclo*(Gly-L-Ser-L-Pro-L-Glu)Pb(NO_3_)]·2H_2_O **2**.


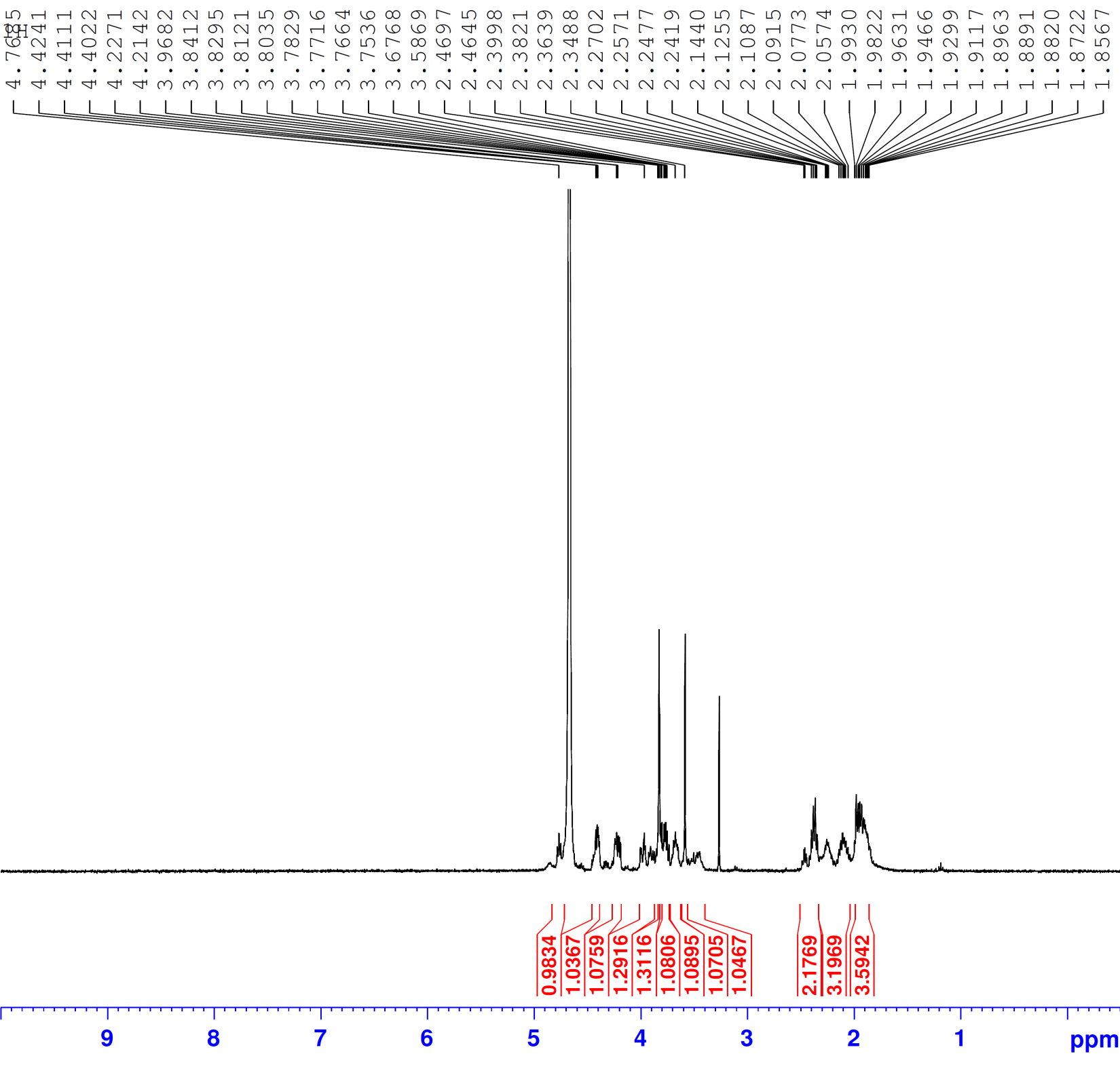


**Figure S3**. ^1^H-NMR spectra of (a) *cyclo*(GSPE) **1**, (b) **1** + Pb^2+^ (1.0 equiv.) in D_2_O at 293 K.


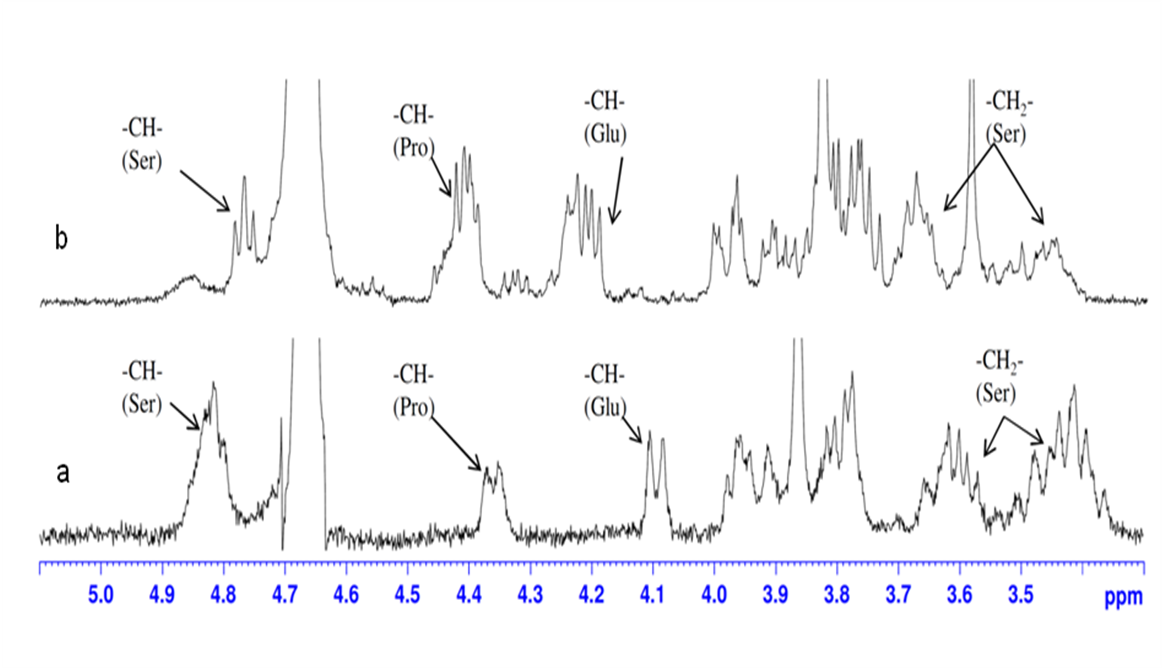


**Figure S4**. MALDI-TOF: *Cyclo*(glycyl-L-seryl-L-prolyl-L-glutamyl) (compound **1**).


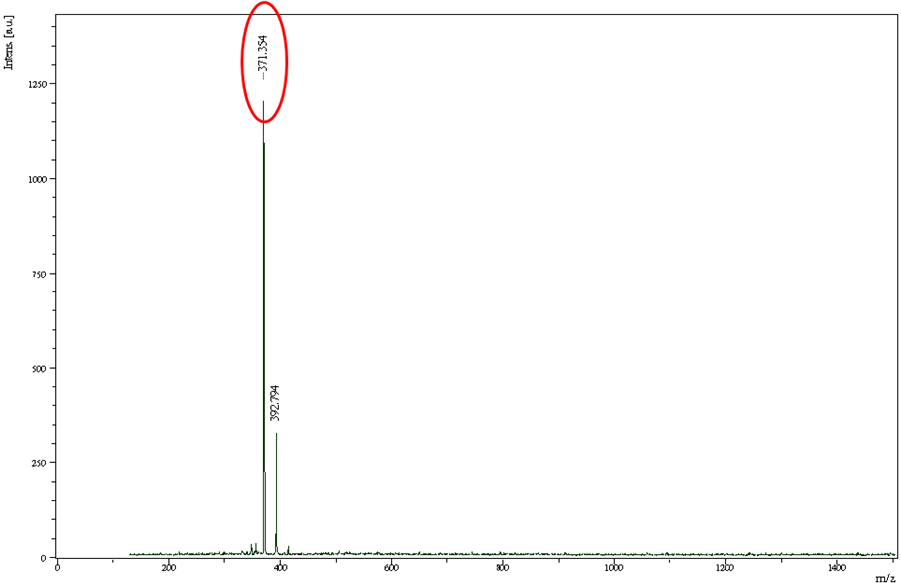


**Figure S5**. ESI-MS: *Cyclo*(glycyl-L-seryl-L-prolyl-L-glutamyl) (compound **1**).


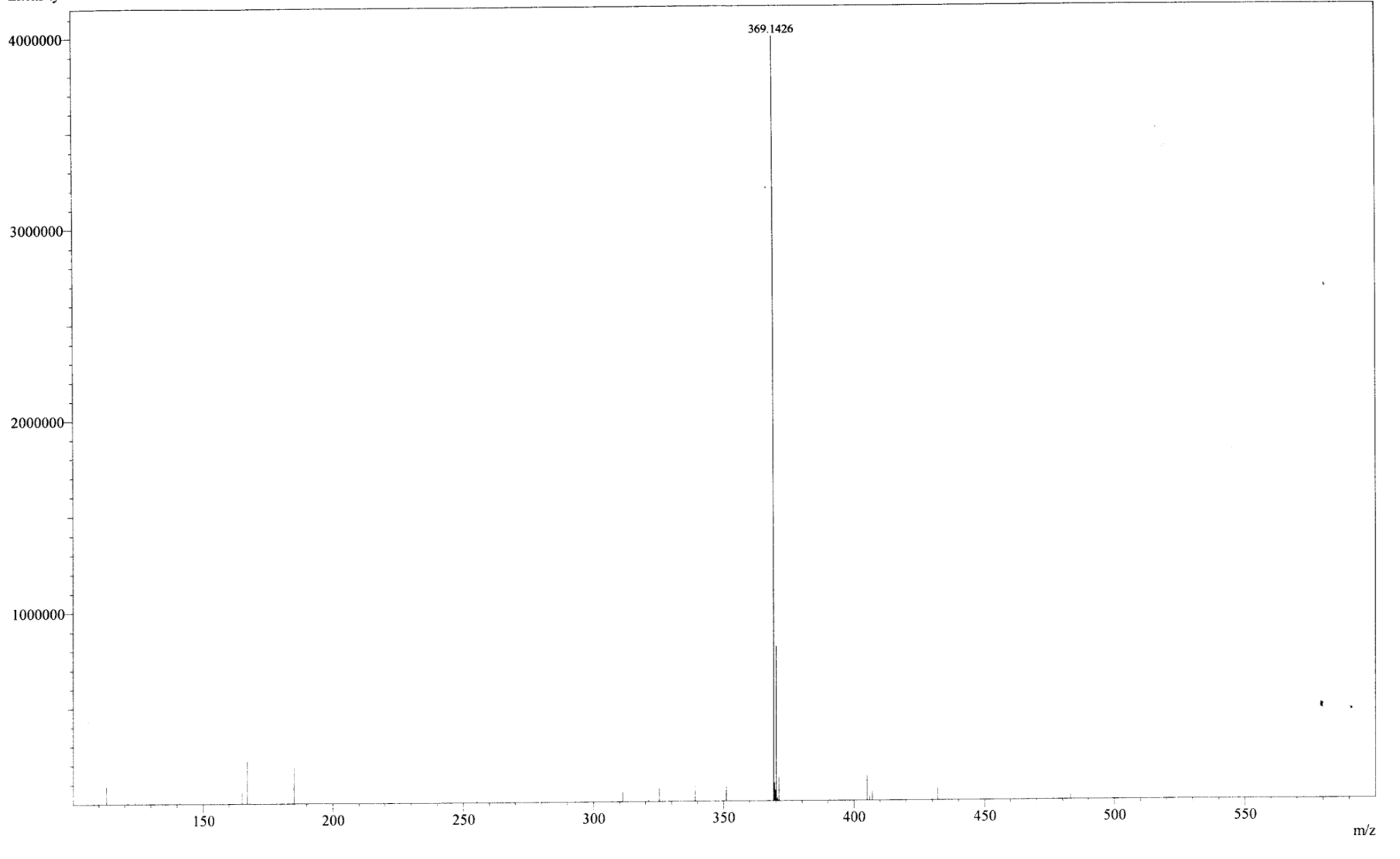


**Figure S6**. MALDI-TOF: On reaction with 1.0 equiv. of Pb^2+^ with *Cyclo*(glycyl-L-seryl-L-prolyl-L-glutamyl): [*cyclo*(Gly-L-Ser-L-Pro-L Glu)Pb(NO_3_)]·2H_2_O **2**.


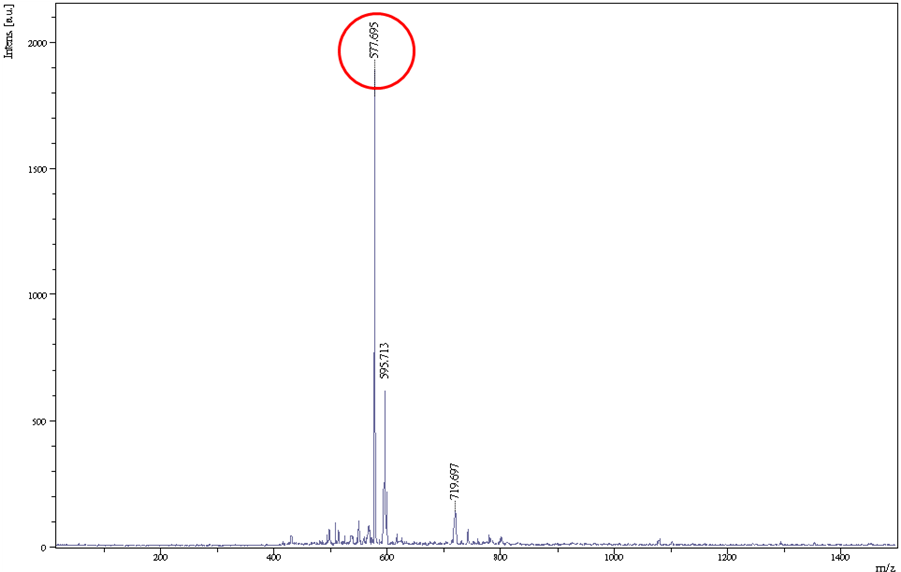


**Figure S7.** (**a,b**) ESI-MS: On reaction with 1.0 equiv. of Pb^2+^ with *Cyclo*(glycyl-L-seryl-L-prolyl-L-glutamyl): [*cyclo*(Gly-L-Ser-L-Pro-L Glu)Pb(NO_3_)]·2H_2_O **2**.


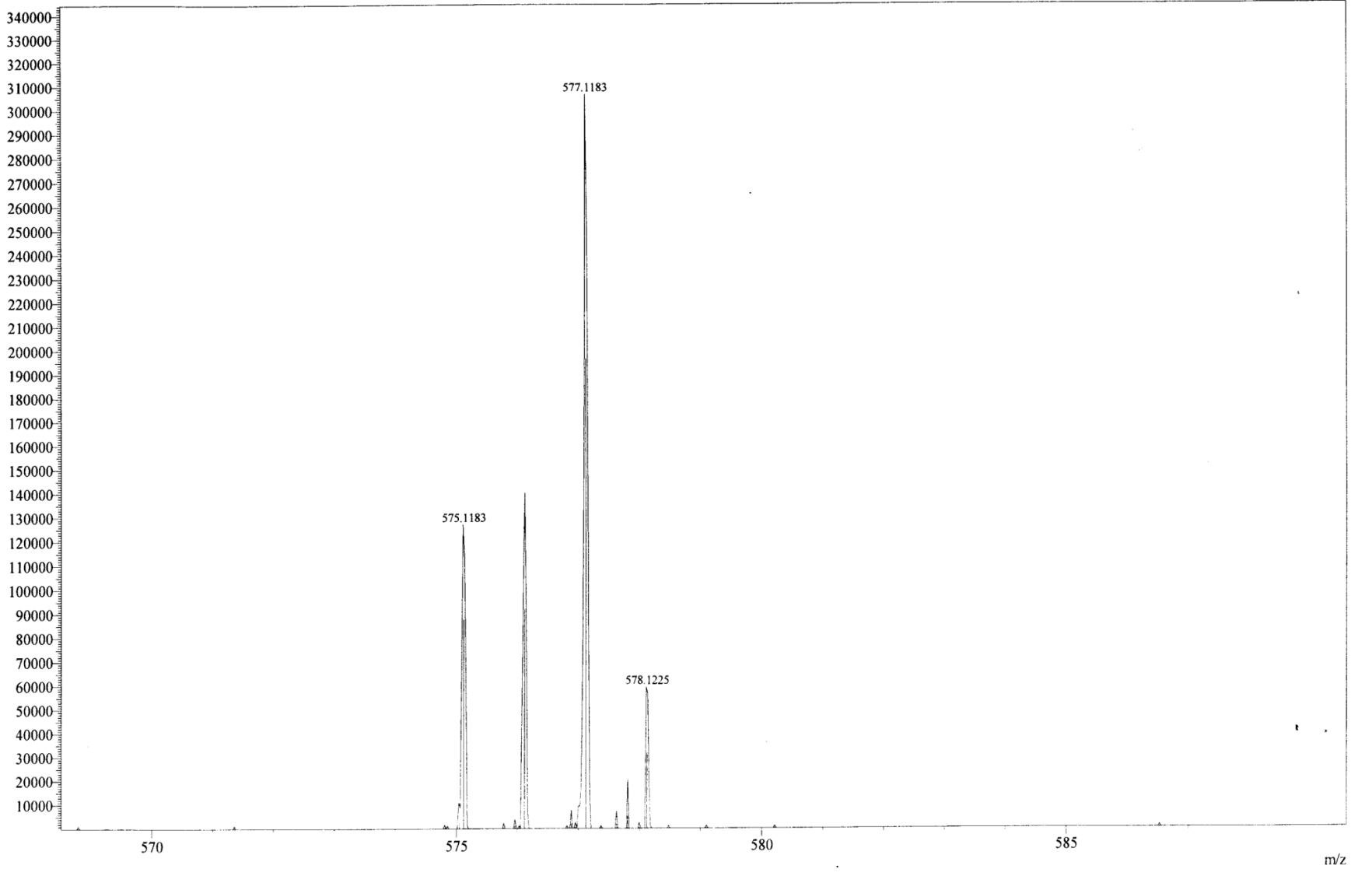


(**a**)

**Figure S7.** *Cont.*


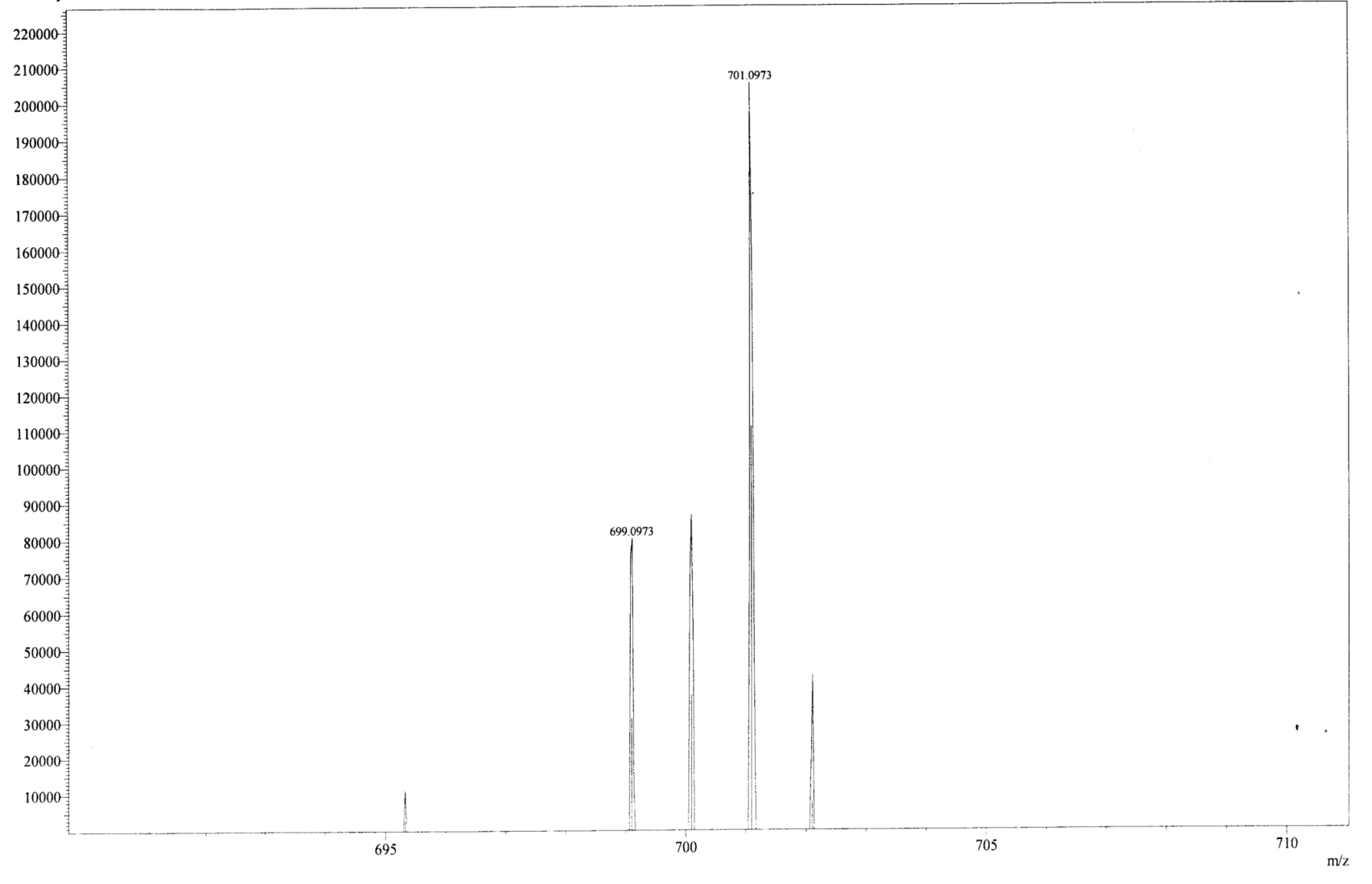


(**b**)

**Figure S8.** IR spectrum of *Cyclo*(glycyl-L-seryl-L-prolyl-L-glutamyl) **1**.


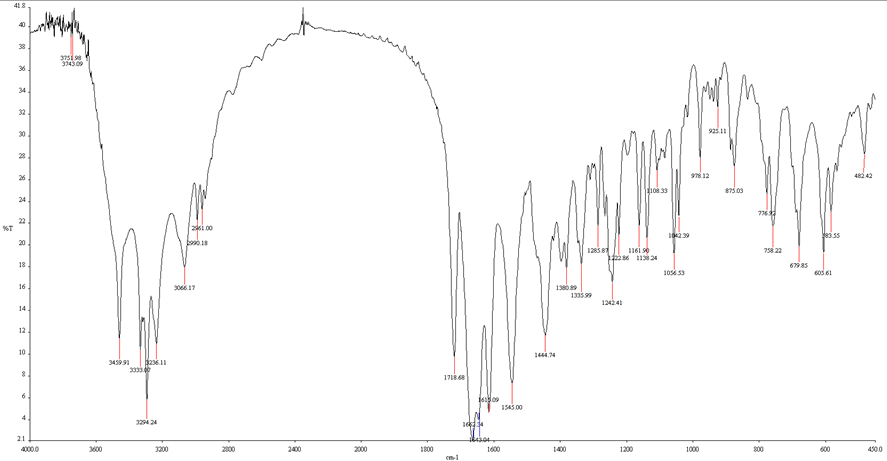


**Figure S9**. IR spectrum of [*cyclo*(Gly-L-Ser-L-Pro-L-Glu)Pb(NO_3_)]·2H_2_O **2**.


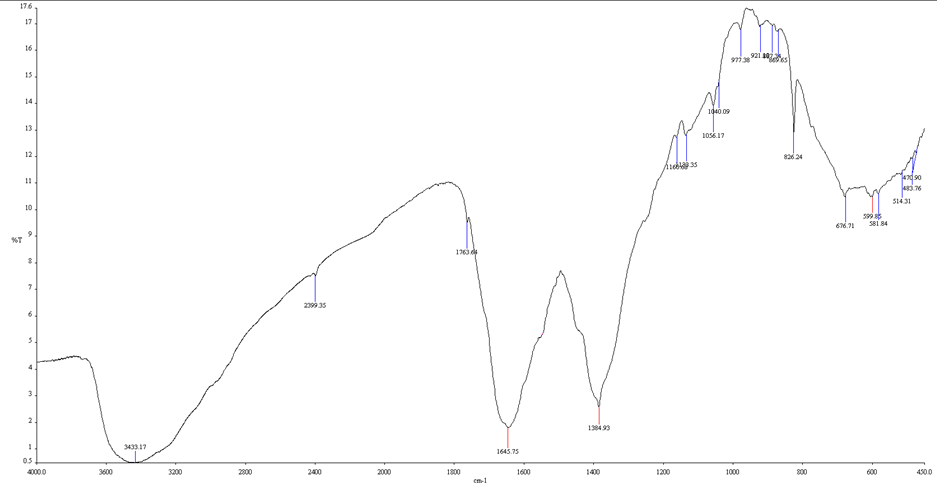

Supplement: Supplementary file 1 [file molecules-18-04972-s001.doc]
